# Preterm birth, unplanned hospital contact, and mortality in infants born to teenage mothers in five countries: An administrative data cohort study

**DOI:** 10.1111/ppe.12685

**Published:** 2020-04-28

**Authors:** Katie Harron, Maximiliane Verfuerden, Ibinabo Ibiebele, Can Liu, Alex Kopp, Astrid Guttmann, Jane Ford, Jan van der Meulen, Anders Hjern, Ruth Gilbert

**Affiliations:** ^1^ Institute of Child Health University College London London UK; ^2^ Royal North Shore Hospital The University of Sydney Northern Clinical School Sydney NSW Australia; ^3^ Department of Public Health Sciences Centre for Health Equity Studies (CHESS) Stockholm University Stockholm Sweden; ^4^ The Institute for Clinical Evaluative Sciences Toronto ON Canada; ^5^ London School of Hygiene and Tropical Medicine London UK

**Keywords:** adolescence, hospitalisation, infant mortality, infant, newborn, maternal age, preterm delivery

## Abstract

**Background:**

Young maternal age is associated with lower birthweight and higher rates of preterm birth and childhood hospitalisations. Internationally, teen pregnancy rates vary widely, reflecting differences in social, welfare, and health care factors in different cultural contexts.

**Objectives:**

To determine whether the increased risk of adverse infant outcomes among teenage mothers varies by country, reflecting different national teenage birth rates and country‐specific social/welfare policies, in Scotland (higher teenage pregnancy rates), England, New South Wales (NSW; Australia), Ontario (Canada), and Sweden (lower rates).

**Methods:**

We used administrative hospital data capturing 3 002 749 singleton births surviving to postnatal discharge between 2010 and 2014 (2008‐2012 for Sweden). We compared preterm birth (24‐36 weeks’ gestation), mortality within 12 months of postnatal discharge, unplanned hospital admissions, and emergency department visits within 12 months of postnatal discharge, for infants born to mothers aged 15‐19, 20‐24, 25‐29, and 30‐34 years.

**Results:**

Compared to births to women aged 30‐34 years, risks of adverse outcomes among teenage mothers were higher in all countries, but the magnitude of effects was not related to country‐specific rates of teenage births. Teenage mothers had between 1.2% (95% confidence interval [CI] 0.7, 1.7, Sweden) and 2.0% (95% CI 1.4, 2.5, NSW) more preterm births, and between 9.8 (95% CI 7.2, 12.4, England) and 19.7 (95% CI 8.7, 30.6, Scotland) more deaths per 10 000 infants, compared with mothers aged 30‐34. Between 6.4% (95% CI 5.5, 7.4, NSW) and 25.4% (95% CI 24.7, 26.1, Ontario), more infants born to teenage mothers had unplanned hospital contacts compared with those born to mothers aged 30‐34.

**Conclusions:**

Regardless of country, infants born to teenage mothers had universally worse outcomes than those born to older mothers. This excess risk did not vary by national rates of livebirths to teenage mothers. Current mechanisms to support teenage mothers have not eliminated maternal age‐related disparities in infant outcomes; further strategies to mitigate excess risk in all countries are needed.

**Synopsiss**

1 **Study question**

Does the effect of teenage motherhood vary in settings with different teenage pregnancy rates and policy contexts?

2 **What's already known**

Children born to teenagers have greater health care needs than those born to older mothers. Social risk factors associated with teenage motherhood are likely to become more concentrated where teenage motherhood is less common.

3 **What this study adds**

We highlight a consistent, substantially increased risk of adverse birth and infant outcomes associated with teenage motherhood, but no clear patterns according to country‐specific rates of births to teenage mothers or to relative disadvantage for teenage mothers. Our cross‐national cohort suggests that the specific needs of teenage mothers and their infants are not currently being met by public health programmes in a range of jurisdictions.

## 1 BACKGROUND

Children born to teenage mothers (aged < 20 years) have greater health care needs than those born to mothers in their 20s and older, and evidence suggests that social determinants explain much of the excess risk of adverse outcomes.^1^, ^2^, ^3^ Social, welfare, and health care factors influence outcomes of teenage pregnancies by determining who gets pregnant and who chooses to proceed with their pregnancy and by affecting the health of young mothers prior to and during pregnancy. For example, a UK survey found that only 12% of teenage pregnancies were planned, compared with two‐thirds of pregnancies in mothers aged 30‐34 years.^4^ The adverse circumstances associated with young maternal age and unplanned pregnancies (eg drug or alcohol abuse and smoking) are also damaging to the health of the mother and baby. Such risky behaviours during pregnancy can lead to adverse neonatal outcomes, which in turn increase health care needs and use of health services throughout childhood and beyond.

Teenage pregnancy rates vary widely even among high‐income countries, reflecting differences in the social, welfare, and health care factors that influence choice about pregnancy among young women and predispose teenagers to pregnancy in different contexts.^5^ Social risk factors such as deprivation and opportunities, aspirations, and expectations for education or employment that are associated with teenage motherhood are likely to become more concentrated in settings where teenage motherhood is more stigmatised and less common or where teenage pregnancies are concentrated in historically marginalised groups. For example in Canada and Australia, Indigenous women have the highest rates of teenage pregnancies and also have a range of other historical social risk factors that disadvantage Indigenous communities and are associated with adverse maternal and infant outcomes.^6^, ^7^ In Sweden, the magnitude of associations between teenage motherhood and criminal convictions, poor academic performance, and substance‐related problems have intensified as teenage pregnancy rates have fallen over time.^8^ Similarly in Canada, falling teenage pregnancy rates have been less pronounced in teenagers with mental illness.^9^ This suggests that as teenage pregnancy rates decline, teenagers who do give birth represent a more vulnerable group, with a higher concentration of individual risk factors such as mental health, conduct problems, or adversity.^10^

Provided careful consideration is given to the comparability and quality of data collected in different contexts, international comparisons can offer powerful external benchmarks and evidence on where and how improvements to health and services might be made.^2^ In this study, we explored variation in the relationship between teenage motherhood and infant outcomes across a range of different societal contexts. Our hypothesis was that the magnitude of difference between teenage and older mothers would vary according to the relative disadvantage for teenage mothers and according to universality of preventive strategies, antenatal care, and access to services. We expected to see a stronger effect of teenage motherhood in low‐prevalence settings or in settings where young motherhood predominates in low socio‐economic groups.

We created birth cohorts from linked administrative data in five countries, where the number of livebirths per 1000 females aged 15‐19 varied between 23 (Scotland), 21 (England), 16 (Australia), 13 (Canada), and 2 (Sweden).^5^ We focussed on clinically important infant outcomes known to be related to social disadvantage and to health behaviours prior to and during pregnancy (preterm birth and mortality). We also compared measures of service need (unplanned hospital admissions and emergency department visits) that could be amenable to support for parenting and access to postnatal health care for mothers and children after discharge from hospital following delivery. Specialised health programmes to support infant care by young or vulnerable mothers exist in each of these jurisdictions, but differ in the ways in which early support is targeted (Box S1).

## 2 METHODS

We obtained data from five countries to create a population‐based cohort study of babies and their mothers in Scotland, England, New South Wales, (NSW; Australia), Ontario (Canada), and Sweden (Tables S1 and S2). Unplanned inpatient admissions and emergency department (ED) visits were extracted from encoded hospital records collected for reimbursement or administrative purposes.

### 2.1 Cohort selection

We included singleton infants born to mothers aged <35 years between April 2010 and March 2014 (April 2008‐2012 for Sweden), whose death or hospital contacts within 12 months from postnatal discharge were captured in the data. Infants born to mothers aged ≥35 years were excluded, as adverse outcomes for this group might relate to biological age (rather than the social risk factors that are less likely to be amenable to intervention). We restricted our cohort to infants who were alive at postnatal discharge, to avoid bias due to differences in obstetric and neonatal care quality between countries (eg delivery and treatment for infants with congenital anomalies or severe birth defects). Due to expected differences in definitions for viability between countries, we excluded infants born <24 weeks of completed gestation.^11^ We excluded infants with incomplete data on maternal age, gestational age at birth, or socio‐economic status (Table S3a,b).

### 2.2 Exposures

Our main exposure variables were maternal age, gestational age, and socio‐economic status (SES) quintile. Maternal age was categorised as 15‐19 (teenage mothers), 20‐24, 25‐29, and 30‐34 years. Gestational age was based on best estimates from menstrual dates or ultrasound and categorised as 24‐27, 28‐31, 32‐33, 34‐36, 37‐38, or ≥39 weeks.

In Scotland, England, and New South Wales, SES was determined using indices incorporating multiple measures of deprivation, including income, unemployment, educational attainment, and car ownership. In Ontario and Sweden, SES was based on income alone (Table S2). Quintiles were based on the whole population except for Sweden, where quintiles were based on the cohort of mothers delivering in each year. We did not assume that measures of SES were directly comparable between countries or that these measures would adequately control for individual socio‐economic position, but included SES quintile to understand the relative disadvantage of teenage mothers *within* each country.

We included only a limited number of covariates in the analysis for two reasons. First, we wanted to avoid assumptions about similarity of coding between countries (eg identification, timing, and coding of congenital anomalies) and therefore did not investigate any diagnostic information. Second, permissions in each country restricted the sharing of individual‐level data, and so, we were required to use aggregate data without small cell sizes. We derived postnatal length of stay (by gestational age), to explore whether the intensity of health care support immediately following birth explained any differences between countries (Table S4).

### 2.3 Outcomes

We chose outcomes known to be related to sociodemographic disadvantage before and after birth and to health behaviours prior to and during pregnancy. We derived the number of infants who were born preterm (<37 completed weeks of gestation), and the number who died, or had ≥1 unplanned inpatient admission (for any cause) or ED visit within 12 months of postnatal discharge (ie all infants had the same time at risk, irrespective of postnatal hospital stay). Admissions were defined as unplanned based on the method of admission coded within hospital records (excluding “planned” or “booked” admissions; transfers were not counted as new admissions). ED data were not available for Scotland.

### 2.4 Statistical analysis

We analysed the association between maternal age and infant outcomes using generalised linear models with log link or identity link to generate relative risks (RR) and risk differences (RD), respectively. Mothers aged 30‐34 years served as the reference group. We included interaction terms for teenage motherhood and country in order to determine whether the effect of teenage motherhood differed between countries. We then visually inspected plots of effect sizes to determine whether any variation between countries was related to national rates of livebirths to teenage mothers (Table S1).

For modelling hospital admissions or ED visits, infants who died in the 12 months following postnatal discharge were treated as having the outcome (as death was a competing risk for hospital contact). We modelled the percentage of infants with ≥1 emergency hospital contact of any kind (ie unplanned admission or ED), since previous evidence demonstrates substantial differences in thresholds for admission from ED across jurisdictions.^12^ For this outcome, we compared unadjusted RRs with RRs adjusted for SES and median postnatal length of stay. We used multilevel models to allow the effect of risk factors to differ across countries (including random slopes for SES). Analyses were conducted in stata 15 (StataCorp LP).

### 2.5 Missing data and sensitivity analyses

We conducted a sensitivity analysis including infants who did not survive to postnatal discharge in the English data. To explore the effect of missing data on gestational age and SES, we conducted a sensitivity analysis using multiple imputation on the English data (Table S5).

### 2.6 Ethics approval

NSW: Approval was obtained from NSW Population and Health Services Research Ethics Committee (2012/12/430). England: Approval for HES was obtained from NHS Digital; the analysis of anonymous data was exempt from UK NREC approval. Ontario: Use of ICES data was approved by the institutional review board at Sunnybrook Health Sciences Centre, Toronto.

## 3 RESULTS

**Figure 1:**
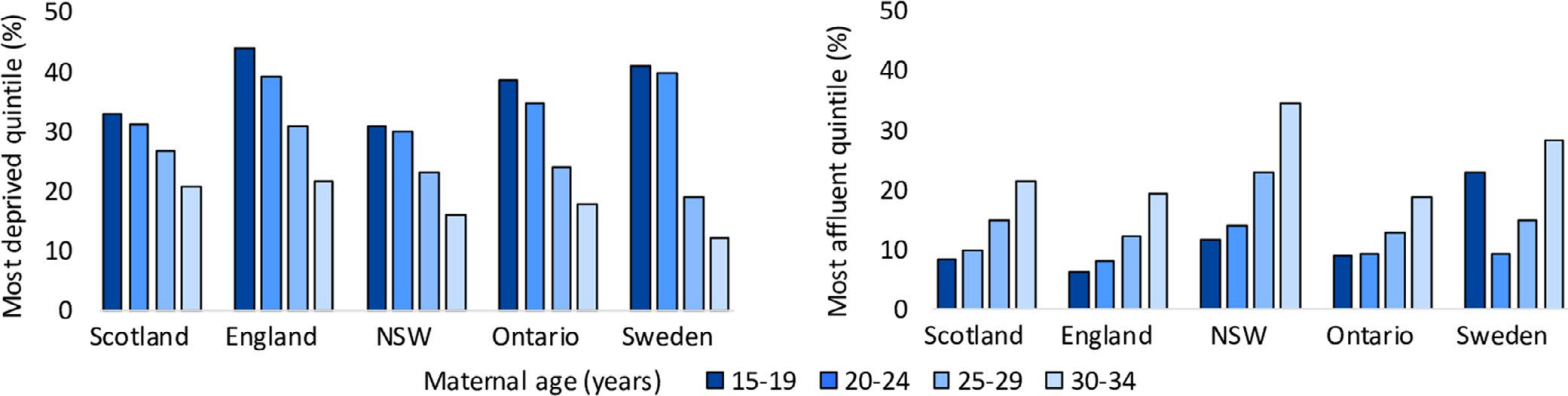
Percentage of mothers in most deprived and most affluent quintiles* by maternal age and country. *Quintiles are based on whole population except for Sweden, where quintiles are based on the study population

Characteristics of the study population are shown in Table 1. In all countries, teenage mothers were more likely to fall within the most deprived quintile of their population than older mothers (Figure 1). The magnitude of socio‐economic differences varied across countries, with largest disparities observed in England and Ontario. In Sweden, the higher proportion of teenage mothers within the most affluent quintile likely reflects those still living at home with parents (SES for Sweden was based on household income rather than neighbourhood measures).

**Table 1:** Study population characteristics: infants born ≥24 wk gestation between 2010 and 2014 (2008‐2012 for Sweden) and surviving to postnatal discharge

	Scotland N = 173 316 No. (%)	England N = 1 812 784 No. (%)	NSW N = 231 306 No. (%)	Ontario N = 472 151 No. (%)	Sweden N = 313 192 No. (%)
Maternal age (y)					
15‐19	12 123 (7.1)	109 990 (6.1)	8839 (3.8)	17 797 (3.8)	6058 (1.9)
20‐24	39 005 (22.7)	414 800 (22.9)	37 559 (16.2)	73 387 (15.5)	53 084 (16.9)
25‐29	59 281 (34.3)	631 949 (34.9)	81 812 (35.4)	168 442 (35.7)	116 138 (37.1)
30‐34	62 907 (35.9)	656 045 (36.2)	103 096 (44.6)	212 525 (45.0)	137 912 (44.0)
Quintile of deprivation^a^					
Most deprived	44 536 (26.0)	549 232 (30.3)	49 381 (21.3)	110 916 (23.5)	62 631 (20.0)
2	37 955 (21.9)	416 681 (23.0)	32 275 (14.0)	98 656 (20.9)	62 663 (20.0)
3	34 961 (19.7)	329 807 (18.2)	45 737 (19.8)	96 585 (20.5)	62 586 (20.0)
4	29 040 (16.7)	273 082 (15.1)	42 966 (18.6)	96 540 (20.4)	62 669 (20.0)
Most affluent	26 797 (15.6)	243 982 (13.5)	60 947 (26.3)	69 454 (14.7)	62 643 (20.0)
Gestational age (wk)					
24‐27	265 (0.1)	2968 (0.2)	351 (0.2)	836 (0.2)	250 (0.1)
28‐31	987 (0.5)	8980 (0.5)	934 (0.4)	2076 (0.4)	632 (0.2)
32‐33	1093 (0.6)	11 560 (0.6)	1372 (0.6)	2835 (0.6)	922 (0.3)
34‐36	6905 (4.0)	72 021 (4.0)	9418 (4.1)	21 104 (4.5)	8916 (2.8)
37‐38	28 047 (16.3)	315 477 (17.4)	54 623 (23.6)	119 697 (25.4)	55 987 (17.9)
≥39	136 019 (78.5)	1 401 778 (77.3)	164 608 (71.2)	325 603 (69.0)	246 485 (78.7)

^a^ Quintiles are based on the whole population except for Sweden, where quintiles are based on the study population.

**Figure 2:**
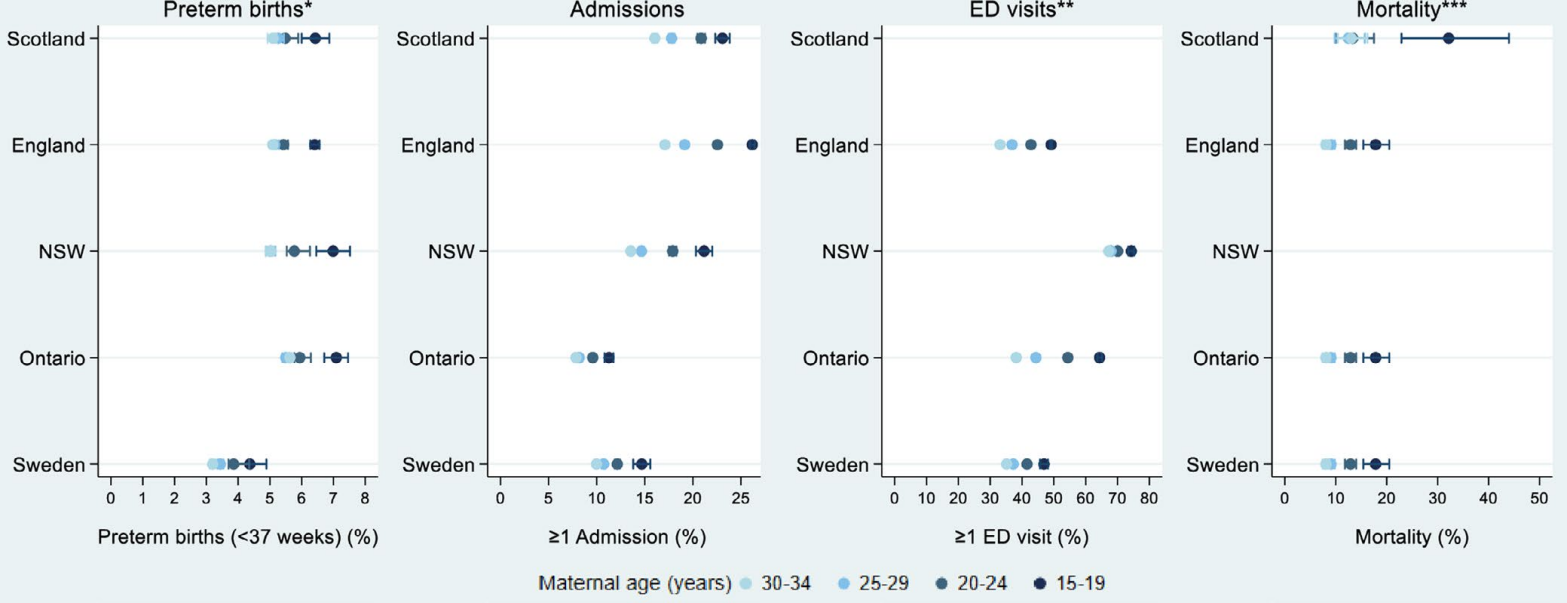
Rates of preterm births*, unplanned hospital admission, emergency department visits, and mortality within 12 mo of postnatal discharge, by maternal age and country. Error bars are 95% confidence intervals. *The preterm birth rate denominator is survivors to discharge home. **ED data were not available in this study for Scotland. ***Cell sizes <10 were suppressed

In all countries, infants born to teenage mothers experienced higher rates of preterm births, unplanned admissions, ED visits, and mortality compared with older mothers (Figure 2, Table S6a‐d). There was an inverse relationship between maternal age and risk of hospital admissions and ED visits, whereby risk of hospital contact increased with decreasing maternal age, and infants born to mothers aged 20‐24 and 25‐29 also had greater health care contacts than those born to mothers aged 30‐34 (Figure 2, Table S6a‐d). However, the relationship between maternal age and mortality or preterm birth did not follow a smooth pattern; there was a larger difference in the risk of outcomes comparing infants born to teenage mothers with those born to mothers aged 20‐24, than for any of the other adjacent age groups (Figure 2, Table S6a‐d).

**Figure 3:**
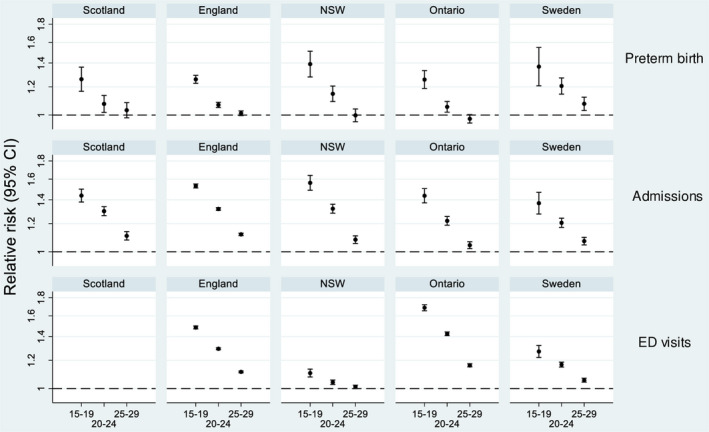
Risk ratios for preterm birth (Preterm birth rate denominator is survivors to discharge home), unplanned admissions, and ED visits. Mothers aged 30‐34 y serve as the reference group. Dashed line indicates no difference. Risk ratios for mortality are provided in Figure S1

**Figure 4:**
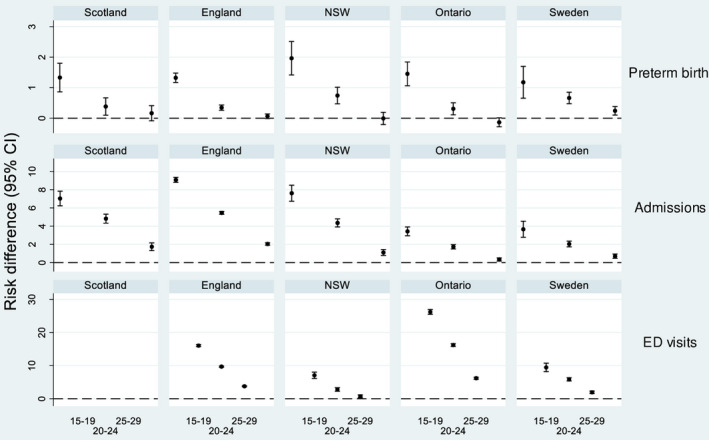
Risk differences for preterm birth (Preterm birth rate denominator is survivors to discharge home), unplanned admissions, and ED visits. Mothers aged 30‐34 y serve as the reference group. Dashed line indicates no difference

Mothers aged 15‐19 were between 1.26 and 1.39 times more likely to have a preterm birth compared with mothers aged 30‐34 (Figure 3, Table 2). This corresponded to 1%‐2% more preterm births for teenage mothers (Figure 4). Both relative and absolute risks for preterm birth were consistent between countries (maternal age‐country interaction *P*‐value = .32 for relative and .16 for absolute risks).

**Table 2:** Risk differences and risk ratios for preterm births^a^, mortality, unplanned admissions, and ED visits within 12 mo of postnatal discharge, comparing mothers aged 15‐19 y with mothers aged 30‐34 y

	Absolute risk N (N per 100 infants or per 10 000 for mortality); all ages	Risk difference per 100 infants or per 10 000 for mortality (95% CI)	Risk ratio (95% CI)
Preterm birth (<37 wk)			
Scotland	9250 (5.3)	1.3 (0.9, 1.8)	1.26 (1.17, 1.36)
England	25 529 (5.3)	1.3 (1.2, 1.5)	1.26 (1.23, 1.29)
NSW	12 075 (5.2)	2.0 (1.4, 2.5)	1.39 (1.28, 1.51)
Ontario	26 851 (5.7)	1.5 (1.1, 1.8)	1.26 (1.19, 1.33)
Sweden	10 720 (3.4)	1.2 (0.7, 1.7)	1.37 (1.21, 1.55)
Mortality^b^			
Scotland	247 (14.3)	19.7 (8.7, 30.6)	2.47 (1.69, 3.61)
England	1831 (10.1)	9.8 (7.2, 12.4)	2.21 (1.88, 2.61)
NSW	—	—	—
Ontario	389 (8.2)	16.7 (9.7, 23.8)	3.92 (2.74, 5.60)
Sweden	163 (5.2)	12.7 (2.5, 23.0)	4.38 (2.23, 8.61)
% of infants with ≥1 unplanned admission			
Scotland	31 577 (18.2)	7.0 (6.2, 7.8)	1.44 (1.38, 1.50)
England	355 574 (19.6)	9.1 (8.8, 9.4)	1.53 (1.51, 1.55)
NSW	34 562 (14.9)	7.6 (6.7, 8.5)	1.56 (1.49, 1.64)
Ontario	39 521 (8.4)	3.4 (2.9, 3.9)	1.44 (1.37, 1.50)
Sweden	33 560 (10.7)	3.7 (2.8, 4.5)	1.37 (1.28, 1.47)
% of infants with ≥1 ED visit^c^			
Scotland	—	—	—
England	681 708 (37.6)	16.0 (15.7, 16.4)	1.48 (1.47, 1.50)
NSW	157 822 (68.2)	7.1 (6.1, 8.0)	1.11 (1.08, 1.13)
Ontario	207 221 (43.9)	26.2 (25.5, 26.9)	1.69 (1.65, 1.72)
Sweden	116 645 (37.2)	9.5 (8.2, 10.7)	1.27 (1.22, 1.32)
% of infants with ≥1 hospital contact (unplanned admission, ED visit or mortality)^c^			
Scotland	—	—	—
England	779 824 (43.0)	16.2 (15.8, 16.5)	1.42 (1.41, 1.43)
NSW	160 029 (69.2)	6.4 (5.5, 7.4)	1.09 (1.07, 1.12)
Ontario	212 786 (45.1)	25.4 (24.7, 26.1)	1.64 (1.61, 1.67)
Sweden	127 424 (40.7)	9.8 (8.5, 11.0)	1.25 (1.21, 1.30)

^a^ The denominator for preterm births was survivors to discharge home.

^b^ Cell sizes <10 were suppressed.

^c^ ED data were not available in this study for Scotland.

Other than in NSW, risk of mortality increased by between 2.21 and 4.38 times for teenage mothers compared with mothers aged 30‐34 (Table 2, Figure S1). There was some evidence for differences in relative risks between countries (*P*_interaction_ = .04). Absolute differences were similar across countries (*P*_interaction_ = .09) and ranged from 9.8 to 19.7 per 10 000 babies (Figure S2).

Infants of teenage mothers were between 1.37 and 1.56 times more likely to have ≥1 unplanned admission compared with mothers aged 30‐34 (Table 2, Figure 3). Both relative and absolute effects of maternal age on unplanned admissions differed between countries (*P*_interaction_ = .007 and <.001).

Risk ratios for ED visits were substantially larger in Ontario and England than in NSW or Sweden (Figure 3). Risk differences followed a similar pattern (Figure 4), and there was evidence of differences in both absolute and relative risks between countries (*P*_interaction_ < .001).

The effect of maternal age on any outcome was not related to national rates of births to teenage mothers (highest for Scotland and England, and lowest for Sweden) nor to absolute risk levels of the outcomes (Figures 3 and 4; Table 2). Adjusting for SES slightly attenuated the increased risk of any unplanned hospital contact (admission, ED or mortality) for teenage mothers in England, Ontario, and Sweden (Figure S3). Adjusting for median postnatal length of stay had no effect on model estimates.

Sensitivity analyses using multiple imputation for missing values in the English data (Table S5) and including infants who did not survive to postnatal discharge (Table S7) provided results consistent with the main analysis.

## 4 COMMENT

### 4.1 Principal findings

This study demonstrates an inverse relationship between maternal age and adverse infant outcomes, with a substantially increased risk of adverse outcomes for infants born to teenage mothers across five high‐income settings. The twofold to fourfold increased risk of mortality in the 12 months postnatal discharge for teenage compared with older mothers was consistent and striking in all countries (except for NSW, where there were too few mortality events to compare rates), corresponding to between 10 and 20 more deaths per 10 000 infants born to teenage mothers. The 25%‐40% increased risk of preterm birth for teenage mothers was also similar between countries, corresponding to an additional 1%‐2% of preterm births compared with mothers aged 30‐34 years. There were substantial differences between countries in the effect of teenage motherhood on unplanned admissions (40%‐60% increased risk) and on ED visit rates (11%‐70% increased risk) compared with infants born to mothers aged 30‐34 years.

### 4.2 Strengths of the study

Strengths include the large sample size covering a range of contexts and comparability of hospital data captured in different settings. We purposefully chose outcomes unaffected by differences in coding practice and considered inpatient admissions and ED visits separately and together, based on previous evidence of differences in thresholds for admission and health‐seeking behaviours between countries.^12^ By comparing within‐country risks according to maternal age, we avoided making inferences about differences in absolute outcomes between countries, which might have reflected differences in obstetric or neonatal care quality or different admission policies.

### 4.3 Limitations of the data

We were unable to access data on primary care or community services. In order to avoid comparisons of neonatal care between countries, we focussed on outcomes known to be related to sociodemographic disadvantage and restricted our cohort to infants surviving to postnatal discharge. However, we assumed that within each country, neonatal care was similar across maternal age groups. Our measure of preterm birth (including only those who survived to postnatal discharge) inhibits comparisons with other studies and may have resulted in an underestimation of the relationship between preterm birth and maternal age (eg if preterm infants who died before discharge were more likely to be born to teenage mothers). However, analyses exploring the breakdown of deaths before discharge by maternal age and country (Table S3) and sensitivity analyses including infants who did not survive to postnatal discharge (Table S5) provided estimates consistent with our main approach.

This study was limited by some methodological difficulties. Firstly, the SES measures are unlikely to capture all social risk factors related to teenage motherhood, since age at childbirth, in and of itself, is a measure of social risk.^13^, ^14^ The relationship between deprivation at the neighbourhood, household, and individual level is complex, particularly for teenage mothers who may live with relatives. For Sweden, we had access only to SES quintiles for the population of mothers included in the cohort; this may have diluted the relationship between SES and teenage pregnancy, as mothers (especially teenagers) are more deprived than the general population.^15^ Secondly, we did not have any information on parity, and it is possible that some of the effect of teenage motherhood can be explained by teenage mothers more often being first‐time mothers than older mothers. However, almost a third of those who fall pregnant as a teenager go on to have a second teenage pregnancy in the UK, and evidence from a number of countries shows that teenage mothers remain at risk of adverse birth outcomes, even in their second pregnancies.^16^

Further limitations include insufficient power to formally examine the relationship between effect sizes and rates of livebirths to teenage mothers in each country (eg using meta‐regression), and suppression of small cell sizes for data protection, which meant that we were unable to investigate additional covariates or causes of admission. Levels of missing data varied by country (Table S4) and may have been related to variables of interest. However, sensitivity analysis using multiple imputation for missing data provided results consistent with the main analysis (Table S5). Finally, a full analysis of policy context of targeted support for young and vulnerable mothers may help explain differences between countries, but was beyond the study scope.

### 4.4 Interpretation

Whilst revealing variation in the effect of teenage motherhood across five high‐income countries, our findings did not demonstrate clear patterns according to rates of livebirths to teenage mothers, relative disadvantage of teenage mothers, or universality of preventive strategies and access to services. Sweden provides a context where primary preventive strategies are successful in reducing teenage pregnancies: teenage mothers in Sweden account for only 1.1% of births compared with 2.8% in Ontario, 3.0% in NSW, 4.6% in England, and 5.4% in Scotland (Table S1). However, the substantial increased risk of preterm birth and postdischarge infant mortality for teenagers who do become mothers in all five countries could indicate that choice about motherhood may be most limited for disadvantaged women who have social risk factors associated both with teenage pregnancy and with adverse birth outcomes. Despite Sweden's low rates of teenage motherhood and smaller social disparities (eg as measured through relative child poverty) compared with the other countries, our ecological analysis suggests that secondary prevention strategies for promoting the health of teenage mothers and their babies in Sweden are no better or worse in relation to countries with greater social disparities and less welfare benefits.^17^ More evidence on the targeting, delivery, and fidelity of specific policies aimed at young or vulnerable mothers (Box S1) is needed to understand the relative effects of these strategies in different contexts.

In all countries, infants born to mothers aged 20‐24 also experienced higher rates of preterm birth, hospital contact, and mortality than older mothers. This sustained gradient effect of maternal age supports previous evidence on the importance of social, economic, and behavioural factors that predispose some young women to pregnancy, rather than biological immaturity, in explaining disparities in infant outcomes for teenage mothers.^18^ It also contributes to the debate on maternal weathering, which posits that lifelong exposure to social and environmental disadvantage leads to broader disparities in health outcomes among disadvantaged populations.^14^, ^19^ Infants born to mothers aged <25 years represent a large proportion of births, particularly in England and Scotland (around 20%, compared with 15% in NSW and Ontario, and 13% in Sweden; Table S1). Policies aiming to reduce and postpone teenage pregnancies are therefore only part of the solution: primary preventive strategies need to address the social, welfare, and health care factors that influence pregnancies among young women.^20^ These same factors are also associated with the health of mothers and their babies, such as lack of education and employment, mental health, trauma history, and substance abuse.^21^ Educational interventions on relationships and access to emergency and long‐acting contraception can help empower young women and promote reproductive choices about timing of pregnancies. For example in England, a multifaceted policy intervention involving health and education agencies has contributed to a decline in teenage and appears to have attenuated the steep deprivation gradient.^13^ Such interventions may also have the potential to help reduce adverse birth outcomes among other young, disadvantaged, or vulnerable women.

Pregnancy and the postnatal period are an important opportunity for secondary preventive approaches to help promote healthy behaviours and to reduce stress, social stigmatisation, and adverse domestic or environmental factors that are damaging to the mother and baby (eg smoking, substance abuse, or domestic violence). Specialised programmes are particularly relevant for teenage mothers who are less likely than older mothers to access antenatal care or care in the community.^22^ Despite early home visiting support being offered universally after birth, with targeted early support recommended for high‐risk groups in all countries (Box S1), the persistence of poorer infant outcomes among teenage mothers suggests that current implementation of targeted services is not effective in meeting the needs of young mothers. Many factors may contribute to this insufficiency, including incomplete coverage and/or voluntary uptake, limited effectiveness for the most disadvantaged mothers, or ineffective implementation of programmes. For example in Sweden, targeted support is recommended for low‐income and immigrant families, yet studies indicate that disadvantaged families do not actually receive the additional support that is recommended.^23^, ^24^ Increased sharing of administrative data between countries and comparisons of national cohorts could help improve our understanding of the best ways to target and deliver preventive strategies aiming to support young mothers and their families during pregnancy and early childhood.

## 5 CONCLUSIONS

Findings from this study reinforce the persistence of disparities in health outcomes among infants born to teenage mothers across multiple countries, irrespective of rates of births to teenage mothers and degree or type of welfare support. Further research is necessary to develop effective public health programmes that support and augment reproductive choice, improve pregnancy and postpartum health by establishing and reinforcing healthy behaviours, and optimise maternal and child health by supporting young and vulnerable families during infancy and early childhood.

## CONFLICT OF INTERESTS

The authors state no conflicts of interest relevant to this work. This work was supported by a Wellcome Trust Sir Henry Wellcome Postdoctoral Fellowship (grant number WT103975). AG is supported by an Applied Chair in Reproductive and Child Health Services and Policy Research from the Canadian Institutes of Health Research. This research was supported in part by the NIHR Great Ormond Street Hospital Biomedical Research Centre and by the Institute for Clinical Evaluative Sciences (ICES) which is funded by an annual grant from the Ontario Ministry of Health and Long‐Term Care (MOHLTC). Parts of this material are based on data and information compiled and provided by the Canadian Institute for Health Information (CIHI) and the Ontario Registrar General (ORG), the original source of which is ServiceOntario. The views expressed are those of the authors and not necessarily those of the NHS, the NIHR, the Department of Health, ICES, CIHI, the Ministry of Government Services, or MOHLTC. The work was supported by the Economic and Social Research Council through the Administrative Data Research Centre for England.

## Supporting information

Supplementary Material
