# Supplementary material for: Preterm birth, unplanned hospital contact, and mortality in infants born to teenage mothers in five countries: An administrative data cohort study
Source: Paediatr Perinat Epidemiol. 2020 Apr 28;34(6):645–54. doi: 10.1111/ppe.12685 (PMC8425326; doi:10.1111/ppe.12685)
Supplement: Supplementary file 1 — Supplementary Material [file PPE-34-645-s001.docx]

# **Supplementary Material**

**Box S1: Specialist programmes for young or vulnerable mothers in each jurisdiction**

**Table S1: Percentage of live births to young mothers (figures based on national published data)**

**Table S2: Description of the data sources**

**Table S3a: Exclusions in by country**

**Table S3b: Exclusions (deaths prior to postnatal discharge) by maternal age and country.**

**Table S4: Postnatal length of stay by gestational age group and country**

**Table S5: Sensitivity analysis using multiple imputation for missing data in the English cohort.**

**Table S6a: Percentage of study population with preterm birth (24-36 completed weeks of gestation), according to country and maternal age**

**Table S6b: Infants with ≥1 unplanned hospital admission in the 12 months following postnatal discharge, according to country and maternal age**

**Table S6c: Infants with ≥1 emergency department visit in the 12 months following postnatal discharge, according to country and maternal age**

**Table S6d: Mortality in infants within the 12 months following postnatal discharge, according to country and maternal age**

**Table S7: Sensitivity analysis including infants who died before postnatal discharge: Risk differences and risk ratios for preterm births, comparing mothers aged 15-19 years with mothers aged 30-34 years.**

**Figure S1: Risk ratios for infant mortality in within 12 months of postnatal discharge comparing infants born to mothers aged 15-19, 20-24, and 25-29 years versus 30-34 years.**

**Figure S2: Risk differences for infant mortality in within 12 months of postnatal discharge comparing infants born to mothers aged 15-19, 20-24, and 25-29 years versus 30-34 years.**

**Figure S3: Crude and adjusted risk ratios for any emergency hospital contact (unplanned admission, emergency department visit or mortality) comparing infants born to mothers aged 15-19 years versus mothers aged 30-34 years.**

**References for Supplementary Material**

**Box S1: Specialist programmes for young or vulnerable mothers in each jurisdiction**

| **Scotland:** NHS Scotland provides a universal health promotion programme known as the Child Health Programme, which includes a structured programme of needs assessment, health promotion, and parenting support provided through contacts with health visitors, school nurses and other health professionals ([https://www2·gov.scot/Resource/0048/00487884·pdf](https://www2.gov.scot/Resource/0048/00487884.pdf)). The programme provides opportunities for health professionals to identify where additional or intensive support is required, and particular attention is recommended for groups such as Looked After Children, homeless families, or families where one or more parent is in prison or is or has been involved with criminal justice services, including parents who have a history of violence, substance misuse, or concerns around mental health. One specific intervention offered to first time teenage mothers is the Family Nurse Partnership (FNP). FNP is a preventive home visiting programme developed to improve pregnancy outcomes, child health and development, and parents’ economic self-sufficiency.^1^ The FNP in Scotland has recently been expanded to enable it to be offered to every eligible teenage mother. |
| --- |
| **England:** Universal maternity services during and post pregnancy are comprehensive and freely available through the Healthy Child Programme (HCP). The frequency of home visits and contact varies according to a proportionate universalism model, meaning that while support is universal, scale and intensity is proportionate to the level of disadvantage.^2^ The National Institute for Health and Care Excellence (NICE) recommends that teenage mothers should be offered age-appropriate services, information about help with transportation to and from appointments, antenatal care in the community, and the opportunity for the partner/father of the baby to be involved in antenatal care (<https://www.nice.org.uk/guidance/CG110/chapter/1-Guidance#young-pregnant-women-aged-under-20>). The HCP also includes a number of preventive interventions, programmes and services targeted at more vulnerable families. Public health commissioners determine which of these ‘progressive’ services are offered, and to whom. As in Scotland, the FNP is aimed at first time, teenage mothers (in 2016, the FNP was offered to around 25-30% of the eligible population in England). |
| **New South Wales:** The NSW Health / Families NSW Supporting Families Early package provides an integrated approach to the care of women, their infants and families in the perinatal period, and offers universal assessment, coordinated care, and home visiting for all parents expecting or caring for a new baby.^3^ The level of care required by a family is categorised according to factors that may impact on ability to parent (unsupported parents, infant care concerns, multiple births, housing, depression and anxiety), or complex risk factors (mental illness, drug and alcohol misuse, domestic violence, or child protection issues. Mothers aged <20 years are identified as a group who may be in need of ongoing support and active follow up. Parents with complex needs are referred to specialist services such as Brighter Futures, which delivers tailored services to families where children are at risk of entering the statutory child protection system.^4^ |
| **Ontario:** Public Health Units offer support for pregnant mothers and families with young children through the Healthy Babies, Healthy Children (HBHC) programme. The programme is free and voluntary, and consists of universal screening with targeted assessments and interventions for families and children from the prenatal period until school age.^5^ The HBHC Postpartum Enhancement aims to achieve a continuum of care from hospital to community through telephone contact and home visits.^6^ HBHC also involves identification of families in need of additional support, targeting mothers aged <22 years of age, newcomer immigrants (who arrived in Canada within three years prior to delivery), mothers with mental health conditions, and those with a history of homelessness, violence, or substance abuse. These mothers are expected to be offered frequent, intensive home visiting with frequency and duration based on the families’ needs. |
| **Sweden:** Child Health Services are offered universally and free of charge to all Swedish parents with children under the age of 6 years, and include home visiting, health examinations and vaccinations, and parenting groups. A home visit is offered when the child is born, and the parents and child go for 14-20 regular and structured visits to the Child Healthcare Centre during the first year.^7^ Child Health Centre nurses have a responsibility to detect children at risk and to pay extra attention to disadvantaged families. Despite recommendations that additional services should be offered to young and single mothers and to low-income or immigrant families, these groups do not appear to actually receive higher levels of intervention.^8^ |

**Table S1: Percentage of live births to young mothers (figures based on national published data)**

|  | **Scotland**  **2010-2014** | **England**  **2010-2014** | **NSW**  **2010-2014** | **Ontario**  **2010-2014** | **Sweden**  **2008-2012** |
| --- | --- | --- | --- | --- | --- |
| % of live births to mothers aged <20 | 5.4 | 4.6 | 3.0 | 2.8 | 1.1 |
| % of live births to mothers aged <25 | 22.9 | 22.5 | 15.9 | 14.6 | 12.8 |
| Live births per 1000 females aged 15-19 (2011)^1^ | 23 | 19 | 16 | 13 | 2 |

*Sources (all last accessed 18/12/19):*

Scotland: Vital Events Reference Tables 2016. Section 3: Births

<https://www.nrscotland.gov.uk/statistics-and-data/statistics/statistics-by-theme/vital-events/general-publications/vital-events-reference-tables/2016/section-3-births>

England: Birth Summary Tables - England and Wales 2016 <https://www.ons.gov.uk/peoplepopulationandcommunity/birthsdeathsandmarriages/livebirths/datasets/birthsummarytables>

NSW: NSW Mother and Babies 2014 and 2016

<https://www.health.nsw.gov.au/hsnsw/Publications/mothers-and-babies-2016.pdf>

Ontario: Live births, by age of mother, Canada, provinces and territories: 2016

https://www150.statcan.gc.ca/t1/tbl1/en/cv.action?pid=1310041601

Sweden: Statistics Sweden: Live births by region, sex and age of mother: [1968 - 2018](http://www.statistikdatabasen.scb.se/pxweb/en/ssd/START__BE__BE0101__BE0101H/FoddaK/)

<http://www.statistikdatabasen.scb.se>

**Table S2: Description of the data sources.** In England, Scotland, and Ontario, cohorts were created using deliveries and births captured in hospital records (births occurring outside of hospital were excluded). In Sweden and NSW, information on home births were included either through information from midwives, or through linking hospital records with birth registrations.

| Scotland | Data were derived from a national electronic birth cohort of children born in Scotland to Scottish resident mothers.^2,3^ Births were identified using National Records for Scotland birth certificates and were linked to maternity, neonatal and administrative hospital records, out-migration, and death certificates (<http://www.adls.ac.uk/nhs-scotland/>). Datasets were linked by the electronic Data Research and Innovation Service (eDRIS: <http://www.isdscotland.org/Products-and-Services/eDRIS/>) using deterministic linkage on the CHI-number, a unique identifier in the Scottish National Health Service. SES was based on the Carstairs index (including car ownership, occupational social class, household overcrowding, and male unemployment.^4^ |
| --- | --- |
| England | Data were extracted from the Hospital Episode Statistics (HES) Admitted Patient Care dataset and the Accident and Emergency dataset.^5,6^ HES is an administrative database collecting detailed information for episodes of care at National Health Service (NHS) hospitals in England, which serves as the basis for a pay-for-performance system of secondary care reimbursement in the NHS internal market (<https://digital.nhs.uk/data-services/hospital-episode-statistics>). SES was based on the Index of Multiple Deprivation.^7^ |
| New South Wales | Data were extracted from linked pregnancy/birth, hospital admission, emergency department and mortality data records.^8^ Pregnancy and birth data were obtained from the Perinatal Data Collection which holds information on all live births and stillbirths of at least 20 weeks gestation or 400 grams occurring in NSW hospitals or homebirth. Hospital admissions data were obtained from the Admitted Patients Data Collection which is a census of all discharges from public, private and day procedure centres in NSW. Emergency department data was obtained from the Emergency Department Data Collection which captures presentations at public hospital emergency departments. Within New South Wales, the majority of emergency care is delivered in public hospitals. Mortality data was obtained from the Register of Births, Deaths and Marriages Death registrations and from the Perinatal Death Review Database. Linkage of the study data was undertaken by the NSW Centre for Health Record Linkage using probabilistic matching methods with reported false positive and false negative rates of 0.5%.^8^ SES was determined from maternal residential postcodes and the Australian Bureau of Statistics Index of Relative Socio Economic Disadvantage scores, based on income, educational attainment, unemployment and car ownership.^9^ |
| Ontario | Data were extracted from linked population-based administrative databases as the Institute for Clinical Evaluative Sciences (ICES) in Toronto.^10^ Hospitalisations data for all admissions to acute care hospitals are collected in the Discharge Abstract Database, and emergency department visits are collected in the National Ambulatory Care Reporting System Database of the Canadian Institute for Health Information (<https://www.cihi.ca/en/discharge-abstract-database-metadata>). Eligible mothers and infants were identified from the Registered Persons Database, which holds information on all Ontario residents with a provincial health care number. Linked maternal and newborn health records were extracted from the MOMBABY dataset, which provides information on all births in hospitals in Ontario and is linked using a unique health care number.^11^ SES was based on neighbourhood income quintile.^12^ |
| Sweden | Data were extracted from the Swedish Medical Birth Register (SMBR), a mandatory database containing details of antenatal, obstetric, and neonatal care for all births in Sweden to resident mothers.^13^ SMBR records were linked with the Swedish Hospital Discharge Register (SHDR) through each child’s study-specific identifier based on their unique personal identity number.^14^ SES was defined as the quintile of disposable household income per person. |

**Table S3a: Exclusions by country.** Categories are not mutually exclusive.

|  |  | **Initial total** | **Maternal age <15** | **Maternal age >=35** | **Gestational age <24 weeks** | **Multiple birth** | **Death before postnatal discharge** | **Missing gestational age, maternal age or SES** | **Other*** | **Total after exclusions** |
| --- | --- | --- | --- | --- | --- | --- | --- | --- | --- | --- |
| **Scotland** | N | 230334 | 48 | 52216 | 55 | 6811 | 183 | 8446 | - | 173316 |
|  | % of initial total |  | 0.02 | 22.7 | 0.02 | 3.0 | 0.08 | 3.7 | - | 74.3 |
| **England** | N | 2562601 | 572 | 510867 | 1710 | 67181 | 17201 | 239479 | - | 1812784 |
|  | % of initial total |  | 0.02 | 19.9 | 0.07 | 2.6 | 0.67 | 9.4 | - | 70.7 |
| **NSW** | N | 392760 | 69 | 92592 | 1318 | 5778 | 3165 | 72864 | 8190 | 231306 |
|  | % of initial total |  | 0.00 | 23.6 | 0.34 | 1.5 | 0.81 | 18.6 | 2.1 | 58.9 |
| **Ontario** | N | 607168 | - | 135017 | 1240 | 24822 | 749 | 31309 | 12005 | 472151 |
|  | % of initial total |  | - | 22.2 | 0.20 | 4.1 | 0.12 | 5.3 | 2.0 | 77.8 |
| **Sweden** | N | 417478 | 19 | 93033 | 130 | 9755 | 73 | 4392 | - | 313192 |
|  | % of initial total |  | 0.00 | 22.3 | 0.03 | 2.3 | 0.02 | 1.0 | - | 75.0 |

* Unlinked data, lost eligibility during follow-up, baby not born in hospital, duplicate records, incomplete follow up, date errors

**Table S3b: Exclusions (deaths prior to postnatal discharge) by maternal age and country.** Data were not available for Sweden.

|  | Scotland^$^ | | England | | NSW | | Ontario** | |
| --- | --- | --- | --- | --- | --- | --- | --- | --- |
| Maternal age (years) | Died  N  (%) | Survived  N  (%) | Died  N  (%) | Survived  N  (%) | Died  N  (%) | Survived  N  (%) | Died  N  (%) | Survived  N  (%) |
| 15-19 | * | * | 710 | 109,990 | 160 | 8679 | 27 | 18,026 |
|  |  |  | 0.64 | 99.36 | 1.81 | 98.19 | 0.15 | 99.85 |
| 20-24 | 13 | 38,992 | 2292 | 414,800 | 408 | 37,151 | 105 | 74,780 |
|  | 0.03 | 99.97 | 0.55 | 99.45 | 1.09 | 98.91 | 0.14 | 99.86 |
| 25-29 | 22 | 59,259 | 3216 | 631,949 | 786 | 81,026 | 203 | 174,890 |
|  | 0.04 | 99.96 | 0.51 | 99.49 | 0.96 | 99.04 | 0.12 | 99.88 |
| 30-34 | 33 | 62,874 | 3167 | 656,045 | 971 | 102,125 | 239 | 221,848 |
|  | 0.05 | 99.95 | 0.48 | 99.52 | 0.94 | 99.06 | 0.11 | 99.89 |

^$^ 97 deaths prior to discharge had missing maternal age

* Supressed due to disclosure control

**Numbers differ from main analysis due to an update of the dataset

**Table S4: Postnatal length of stay by gestational age group and country**

|  | Median postnatal length of stay (days) | | | | |
| --- | --- | --- | --- | --- | --- |
|  | Scotland | England | NSW | Ontario | Sweden |
| Full term | 1 | 1 | 3 | 2 | 2 |
| Early term | 2 | 2 | 3 | 2 | 2 |
| Late preterm | 3 | 4 | 5 | 4 | 4 |
| Moderate preterm | 11 | 19 | 15 | 22 | 21 |
| Very preterm | 21 | 43 | 25 | 45 | 42 |
| Extremely preterm | 37 | 92 | 66 | 92 | 34 |

**Table S5: Sensitivity analysis using multiple imputation for missing data in the English cohort.**

In addition to the 1,812,784 records for England included in the main analysis, an additional 179,139 records were included in the sensitivity analysis for missing data. Of these 179,139 records, 481 were missing both IMD and gestational age, and 168,079 were missing gestational age only. Overall, 9% of records (179,139/1,991,923) had missing values on either gestational age or IMD.

Complete values for gestational age and IMD were imputed using chained equations with 10 imputed datasets in Stata 15. Linear and multinomial logistic regression was used to model the relationship between gestational age and IMD and birth weight, sex, maternal age, multiple births, postnatal length of stay, ethnic group, and outcomes (mortality, unplanned admissions and ED visits within 12 months of postnatal discharge).

|  | **Absolute risk per 100 infants (per 10,000 for mortality; all ages combined)** | **Risk difference per 100 infants**  **(per 10,000 for mortality)** | **95% CI** | **Risk ratio** | **95% CI** |
| --- | --- | --- | --- | --- | --- |
| Preterm birth  (<37 weeks gestation) | 5.6 | 1.1 | 9.7, 13.0 | 1.21 | 1.17, 1.24 |
| Mortality | 10.3 | 10.0 | 7.5, 12.6 | 2.22 | 1.90, 2.59 |
| % of infants with ≥1 unplanned admission | 19.5 | 8.9 | 8.7, 9.2 | 1.52 | 1.50, 1.54 |
| % of infants with ≥1 ED visit | 37.4 | 15.9 | 15.6, 16.2 | 1.48 | 1.47, 1.49 |
| % of infants with ≥1 hospital contact (unplanned admission, ED visit or mortality) | 42.8 | 16.0 | 15.7, 16.3 | 1.42 | 1.41, 1.43 |

**Table S6a: Percentage of study population with preterm birth (24-36 completed weeks of gestation), according to country and maternal age**

|  | **Scotland** | | **England** | | **NSW** | | **Ontario** | | **Sweden** | |
| --- | --- | --- | --- | --- | --- | --- | --- | --- | --- | --- |
|  | N=173,216 | | N=1,812,784 | | N=231,306 | | N=472,151 | | N=317,114 | |
| **Maternal age (years)** | N | % | N | % | N | % | N | % | N | % |
| 15-19 | 780 | 6.4  (6.0-6.9) | 7053 | 6.4  (6.3, 6.6) | 618 | 7.0  (6.5, 7.5) | 1261 | 7.1  (6.7, 7.5) | 265 | 4.4  (3.9, 4.9) |
| 20-24 | 2139 | 5.5  (5.3-5.9) | 22542 | 5.4  (5.4, 5.6) | 2167 | 5.8  (5.5, 6.3) | 4359 | 5.9  (5.8, 6.3) | 2049 | 3.9  (3.7, 4.3) |
| 25-29 | 3121 | 5.3  (5.1-5.4) | 32542 | 5.1  (5.1, 5.3) | 4106 | 5.0  (4.9, 5.2) | 9259 | 5.5  (5.15 5.6) | 3995 | 3.4  (3.3, 3.5) |
| 30-34 | 3210 | 5.1  (4.9-5.2) | 33392 | 5.1  (5.0, 5.2) | 5184 | 5.0  (4.9, 5.1) | 11972 | 5.6  (5.5, 5.7) | 4411 | 3.2  (3.1, 3.3) |
| Total | 9250 | 5.3 | 95529 | 5.3 | 12075 | 5.2 | 26851 | 5.7 | 10720 | 3.4 |

**Table S6b: Infants with ≥1 unplanned hospital admission in the 12 months following postnatal discharge, according to country and maternal age**

|  | **Scotland** | | **England** | | **NSW** | | **Ontario** | | **Sweden** | |
| --- | --- | --- | --- | --- | --- | --- | --- | --- | --- | --- |
|  | N=173,216 | | N=1,812,784 | | N=231,306 | | N=472,151 | | N=317,114 | |
| **Maternal age (years)** | N | % | N | % | N | % | N | % | N | % |
| 15-19 | 2798 | 23.1  (22.2, 23.8) | 28799 | 26.2  (25.9, 26.4) | 1871 | 21.2  (20.3, 22.0) | 2007 | 11.3  (10.8, 11.7) | 890 | 14.7  (13.8, 15.6) |
| 20-24 | 8140 | 20.9  (20.5, 21.3) | 93581 | 22.6  (22.4, 22.7) | 6725 | 17.9  (17.5, 18.3) | 7036 | 9.6  (9.4, 9.8) | 6447 | 12.1  (11.9, 12.4) |
| 25-29 | 10545 | 17.8  (17.5, 18.1) | 120989 | 19.1  (19.0,19.2) | 11994 | 14.7  (14.4, 14.9) | 13797 | 8.2  (8.1, 8.3) | 12458 | 10.7  (10.5, 10.9) |
| 30-34 | 10094 | 16.0  (15.8, 16.3) | 112205 | 17.1  (17.0, 17.2) | 13972 | 13.6  (13.3, 13.8) | 16681 | 7.8  (7.7, 8.0) | 13765 | 10.0  (9.8, 10.1) |
| Total | 30610 | 18.2 | 355574 | 19.6 | 34562 | 14.9 | 39521 | 8.4 | 33560 | 10.7 |

**Table S6c: Infants with ≥1 emergency department visit in the 12 months following postnatal discharge, according to country and maternal age**

|  | **Scotland*** | | **England** | | **NSW** | | **Ontario** | | | **Sweden** | |
| --- | --- | --- | --- | --- | --- | --- | --- | --- | --- | --- | --- |
|  | N=173,216 | | N=1,812,784 | | N=231,306 | | N=472,151 | | | N=317,114 | |
| **Maternal age (years)** | N | % | N | % | N | % | | N | % | N | % |
| 15-19 | - | - | 54057 | 49.1  (48.9, 49.4) | 6569 | 74.3  (73.4, 75.2) | | 11458 | 64.4  (63.7, 65.1) | 2842 | 46.9  (45.7, 48.2) |
| 20-24 | - | - | 177549 | 42.8  (42.7, 43.0) | 26377 | 70.1  (69.6, 70.5) | | 39912 | 54.4  (54.0, 54.7) | 22069 | 41.6  (41.2, 42.0) |
| 25-29 | - | - | 232937 | 36.9  (36.7, 37.0) | 55606 | 68.0  (67.6, 68.3) | | 74719 | 44.4  (44.1, 44.6) | 43258 | 37.2  (37.0, 37.5) |
| 30-34 | - | - | 217165 | 33.1  (33.0, 33.2) | 69336 | 67.3  (67.0, 67.5) | | 81132 | 38.2  (38.0, 38.4) | 48476 | 35.1  (34.9, 35.4) |
| Total | - | - | 681708 | 37.6 | 157822 | 68.2 | | 207221 | 43.9 | 116645 | 37.2 |

* ED data were not available for Scotland in this study

**Table S6d: Mortality in infants within the 12 months following postnatal discharge, according to country and maternal age**

|  | **Scotland** | | **England** | | **NSW*** | | **Ontario** | | **Sweden** | |
| --- | --- | --- | --- | --- | --- | --- | --- | --- | --- | --- |
|  | N=173,216 | | N=1,812,784 | | N=231,306 | | N=472,151 | | N=317,114 | |
| **Maternal age (years)** | N | Rate / 10,000 | N | Rate / 10,000 | N | Rate / 10,000 | N | Rate / 10,000 | N | Rate / 10,000 |
| 15-19 | 39 | 32.2  (22.9, 44.0) | 196 | 17.8  (14.5, 20.5) | - | - | 40 | 22.5  (16.1, 30.6) | 10 | 16.5  (7.9, 30.0) |
| 20-24 | 52 | 13.3  (10.0, 17.5) | 535 | 12.9  (11.8, 14.0) | - | - | 112 | 15.3  (12.6, 18.4) | 35 | 6.6  (4.6, 9.2) |
| 25-29 | 74 | 12.5  (9.8, 15.7) | 572 | 9.1  (8.3, 9.8) | - | - | 115 | 6.8  (5.6, 8.2) | 66 | 5.7  (4.4, 7.2) |
| 30-34 | 82 | 13.0  (10.4, 16.2) | 528 | 8.0  (7.4, 8.8) | - | - | 122 | 5.7  (4.8, 6.9) | 52 | 3.8  (2.8, 4.9) |
| Total | 247 | 14.3 | 1831 | 10.1 | - | - | 389 | 8.2 | 163 | 5.2 |

* Cell sizes <10 were supressed

**Table S7: Sensitivity analysis including infants who died before postnatal discharge: Risk differences and risk ratios for preterm births, comparing mothers aged 15-19 years with mothers aged 30-34 years.**

|  |  | **Absolute risk per 100 infants (all ages combined)** | **Risk difference per 100 infants** | **95% CI** | **Risk ratio** | **95% CI** |
| --- | --- | --- | --- | --- | --- | --- |
| Preterm birth (<37 weeks gestation) | Scotland | 5.4 | 1.3 | 0.9, 1.8 | 1.26 | 1.17, 1.36 |
|  | England | 5.6 | 1.4 | 1.3, 1.6 | 1.27 | 1.24, 1.30 |
|  | NSW | 6.0 | 2.6 | 2.0, 3.2 | 1.45 | 1.34, 1.56 |
|  | Ontario | 5.9 | 1.3 | 1.0, 1.7 | 1.23 | 1.16, 1.30 |
|  | Sweden* | - | - | - | - | - |

*Data not available for Sweden.

**Figure S1: Risk ratios for infant mortality in within 12 months of postnatal discharge comparing infants born to mothers aged 15-19, 20-24, and 25-29 years versus 30-34 years.** Dashed line indicates no difference.

**Figure S2: Risk differences for infant mortality in within 12 months of postnatal discharge comparing infants born to mothers aged 15-19, 20-24, and 25-29 years versus 30-34 years.** Dashed line indicates no difference.

**Figure S3: Crude and adjusted risk ratios for any emergency hospital contact (unplanned admission, emergency department visit or mortality) comparing infants born to mothers aged 15-19 years versus mothers aged 30-34 years.**

**References for Supplementary Material**

1. Sedgh, G., et al. (2015). "Adolescent Pregnancy, Birth, and Abortion Rates Across Countries: Levels and Recent Trends." J Adolesc Health **56**(2): 223-230.
2. Hardelid P, Verfuerden M, McMenamin J, Gilbert R. Risk factors for admission to hospital with laboratory-confirmed influenza in young children: birth cohort study. *Eur Respir J* 2017; **50**(3).
3. Administrative Data Liason Service. NHS Scotland Administrative Data Resources http://www.adls.ac.uk/nhs-scotland/ (accessed 15/01/19).
4. Information Services Division NHS National Services Scotland. The Carstairs and Morris Index 2010, 2017. http://www.isdscotland.org/products-and-Services/GPD-Support/deprivation/carstairs/ (accessed 15/01/19).
5. Herbert A, Wijlaars LPMM, Zylbersztejn A, Cromwell D, Hardelid P. Data Resource Profile: Hospital Episode Statistics Admitted Patient Care (HES APC). *Int J Epidemiol* 2017; **Epub**: doi: 10.1093/ije/dyx015.
6. Harron K, Gilbert R, Cromwell DA, van der Meulen JH. Linking data for mothers and babies in de-identified electronic health data. *PLoS One* 2016; **11**(10): e0164667.
7. Office of the Deputy Prime Minister. The English Indices of Deprivation 2004, 2003. http://www.simonpoulter.co.uk/iod/iodpdf/odpm_urbpol_029534.pdf (accessed 15/01/19).
8. Bentley J, Ford J, Taylor L, Irvine K, Roberts C. Investigating linkage rates among probabilistically linked birth and hospitalization records. *BMC Med Res Methodol* 2012; **12**(1): 149.
9. Australian Bureau of Statistics. Socio-Economic Indexes for Areas (SEIFA): 2011, Catalogue 2033.0.55.001, 2013. http://www.abs.gov.au/AUSSTATS/abs@.nsf/DetailsPage/2033.0.55.0012011?OpenDocument (accessed 15/01/19).
10. Harron K, Gilbert R, Cromwell D, Oddie S, Guttmann A, van der Meulen J. H. International comparison of emergency hospital use for infants: data linkage cohort study in Ontario and England. *BMJ Qual Saf* 2018; **27**(1): 31-9.
11. Ray JG, Urquia ML, Berger H, Vermeulen MJ. Maternal and neonatal separation and mortality associated with concurrent admissions to intensive care units. *CMAJ* 2012; **184**(18): E956-E62.
12. Matheson FI, Dunn JR, Smith KL, Moineddin R, Glazier RH. Development of the Canadian Marginalization Index: a new tool for the study of inequality. *Can J Public Health* 2012; **103**(8 Suppl 2): S12-6.
13. Centre for Epidemiology: The National Board of Health and Welfare. The Swedish Medical Birth Register - a summary of content and quality 2003. https://www.socialstyrelsen.se/Lists/Artikelkatalog/Attachments/10655/2003-112-3_20031123.pdf (accessed 15/01/19).
14. Ludvigsson JF, Andersson E, Ekbom A, et al. External review and validation of the Swedish national inpatient register. *BMC Public Health* 2011; **11**: 450.

1. Robling M. The Building Blocks Trial. Evaluating the Family Nurse Partnership Programme in England: A Randomised Controlled Trial, 2015. <https://www.cardiff.ac.uk/__data/assets/pdf_file/0009/504729/Building-Blocks-Full-Study-Report.pdf> (accessed 15/01/19).

2. Department of Health. Healthy Child Programme. Pregnancy and the first five years of life, 2009. [www.gov.uk/government/uploads/system/uploads/attachment_data/file/167998/Health_Child_Programme.pdf](file:///C:\Users\sejjkh1\AppData\Roaming\Microsoft\Word\www.gov.uk\government\uploads\system\uploads\attachment_data\file\167998\Health_Child_Programme.pdf) (accessed 15/01/19).

3. NSW Government. Policy Directive: Maternal & Child Health Primary Health Care Policy, 2010. <http://www1.health.nsw.gov.au/pds/ActivePDSDocuments/PD2010_017.pdf> (accessed 15/01/19).

4. NSW Government. Program Guidelines for the Brighter Futures Program, 2014. <https://www.facs.nsw.gov.au/__data/assets/pdf_file/0020/321185/tabb_bf_program_guidelines_may_2014.pdf> (accessed 15/01/19).

5. Public Health Ontario. Health Babies Healthy Children: Process Implementation Evaluation, 2014. <https://www.publichealthontario.ca/en/eRepository/HBHC_Executive_Summary_EN_2014.pdf> (accessed 15/01/19).

6. Ministry of Health and Long-Term Care. Postpartum Implementation Guidelines for Healthy Babies Healthy Children Program, 2001. <http://www.health.gov.on.ca/english/providers/pub/child/hbabies/postpartum.html> (accessed 15/01/19).

7. McKee M. For the sake of children: social paediatrics in action. A festschrift in honour of Staffan Janson: Karlstad University, 2012. <https://www.issop.org/wp-content/uploads/2013/12/ESSOP_DOCUMENTS_pdf_Various_for-the-sake-of_children_staffan-janson_2012.pdf> (accessed 15/01/19).

8. Wallby T, Hjern A. Child health care uptake among low-income and immigrant families in a Swedish county. *Acta Paediatr* 2011; **100**(11): 1495-503.
